# Mineral Springs and Health Resorts of California

**Published:** 1890-10

**Authors:** 


MINERAL SPRINGS AND HEALTH RESORTS OF CALIFORNIA, with a complete Chemical Analysis of Every Important Mineral Water in the World. Illustrated. A Prize Essay. Annual Prize of the Medical Society of the State of California. Awarded April 20, 1889. By WINSLOW ANDERSON, M. D., joint editor and publisher of the *Pacific Medical Journal;* Assistant Chair Medical Chemistry and Materia Medica, and Teacher of Chemistry in the Laboratories of the University of California in the Medical and Dental Departments; Active Member of the American Medical Association, and of the Medical Society of the State of California, etc., etc., etc., etc., etc., etc. Pp. xxvii.—384. The Bancroft Company, San Francisco. 1890. Price, $1.50.

A short introductory and a brief review of the theories on the Origin of Mineral Springs, is followed by a chapter on the Medicinal Uses of the Various Mineral Waters.

The author very justly berates all charlatans and quacks who claim for mineral waters a panacea for all "the ills that human flesh is heir to." "On the other hand," the doctor says, it would be quite "as flagrant an error to suppose that all the reputed beneficial effects of mineral waters were only the result of extravagant or interested imaginings." The patient is then advised to first consult his regular physician, and, after a thorough diagnosis of the case, to go to that spring recommended, armed with a letter to the resident physician. The patient should follow implicitly the direction of the resident physician, and never allow his own whims or fancies to dictate to him the course he shall pursue. The restless nature which characterizes the average American is taken into consideration, and the patient is advised to *never* "*rush*" the treatment. We are too liable to think that if two or three glasses of a mineral water per diem will do us good, a half dozen or more ought to help us much more and effect a more rapid cure. A very foolish notion, indeed. Apply the reasoning to other remedies and see how absurd it is.

All we need at American health resorts and mineral watering places is to follow the natural scientific regime which has been worked out for centuries in Europe. There every patient confides in his physician, and medical men abroad value the mineral springs more, apparently, than we do in America.

As the standard of medical education is so much higher in Europe than with us, it is no great surprise that so much confidence should be shown physicians abroad. We do not, in any way, mean to belittle the profession at home, for we have many men who stand among the first of the medical world. Aside from that, it is unquestionable that the standard of medical education at home sadly needs elevating. It is high time that the people arose in their righteous indignation and demanded that the "diploma mills" either closed their doors or raised their standard to such a degree that men, who are a lasting disgrace to the profession, he excluded.

The author makes a very strong plea that rational scientific treatment be pursued at mineral watering places, and that the patient follow implicitly the directions of the physician in charge.

In the chapter on The Therapeutic Action of Mineral Waters on the Human Economy, very much valuable information on the doses and therapeutic action of the various classes of mineral waters is given.

A great many analyses of mineral waters are given, showing Dr. Anderson to be an indefatigable analyst of the California springs.

This work is of great value to the physician and the chemist alike, and deserves a very careful reading by both professions.

